# A synergic effect between CYP2C19*2, CYP2C19*3 loss-of-function and CYP2C19*17 gain-of-function alleles is associated with Clopidogrel resistance among Moroccan Acute Coronary Syndromes patients

**DOI:** 10.1186/s13104-018-3132-0

**Published:** 2018-01-18

**Authors:** Hind Hassani Idrissi, Wiam Hmimech, Nada El Khorb, Hafid Akoudad, Rachida Habbal, Sellama Nadifi

**Affiliations:** 1 Laboratory of Genetics and Molecular Pathology, Medical School, University Hassan II, Casablanca, Morocco; 2 Department of Cardiology, University Hospital Center Ibn Rochd, Casablanca, Morocco; 3 Department of Cardiology, University Hospital Center Hassan II, Fes, Morocco

**Keywords:** Acute Coronary Syndromes, Clopidogrel response, CYP2C19*2, CYP2C19*3, CYP2C19*17, Moroccan population

## Abstract

**Objective:**

The main objective of our study was to investigate the association of CYP2C19*2 and CYP2C19*3 loss-of-function and CYP2C19*17 gain-of-function variants of CYP2C19 gene with Clopidogrel resistance in a sample of Moroccan Acute Coronary Syndromes patients.

**Results:**

Our results showed the existence of a synergic effect between the three alleles, statistically very significant, on Clopidogrel resistance among the treated patients (P = 0.0033). For the three variants of the CYP2C19 gene, the heterozygous and homozygous mutant genotypes were the most frequent among ACS patients (CYP2C19*2: 82.76% GA and 10.35% AA; CYP2C19*3: 76.67% GA and 18.33% AA; CYP2C19*17: 66.67% CT and 18.66% TT). Allelic frequencies were 51.73% vs 48.27% (P < 0.001); 56.67% vs 43.33% (P < 0.001); and 52% vs 48% (P = 0.01) for the mutant and wild type alleles of the CYP2C19*2, CYP2C19*3 and CYP2C19*17 variants respectively. Our results support a role of CYP2C19 gene variants as a potential marker of Clopidogrel response. Understanding the functional and clinical consequences of these variants may help for treating patients more effectively, they could be genetically screened and appropriate dose adjustments could be made on the basis of their CYP2C19 genotype.

**Electronic supplementary material:**

The online version of this article (10.1186/s13104-018-3132-0) contains supplementary material, which is available to authorized users.

## Introduction

Prescribed as dual antiplatelet therapy with aspirin, Clopidogrel is an antiplatelet agent used as a basic treatment among patients with Acute Coronary Syndromes (ACS) or undergoing percutaneous coronary intervention (PCI) [1, 2]. Although its widely described effectiveness, Clopidogrel exhibits a large inter-individual variability of response [3, 4]. Multiple factors may influence the pharmacokinetic and pharmacodynamic effects of the molecule, by affecting its intestinal absorption [SNPs of the multi drug resistance 1 (MDR1) gene] [5, 6], metabolism (SNPs of the hepatic Cyp450 isoenzymes), or preventing its effect on its direct target, the P2Y12 receptor [7–10].

Clopidogrel is administrated as an inactive prodrug that requires several biotransformation steps in order to be active. This process takes place via two sequential hepatic reactions of oxidation, catalyzed by the Cytochrome P450 (CYP450) system [11] especially the CYP2C19 located on chromosome 10 (10q24.1–q24.3) [12, 13].

CYP2C19 is a highly polymorphic locus, with almost 27 variants currently reported, from which only three are with normal activity and one with high activity, the others are with no enzymatic activity [11]. The most described CYP2C19 abnormal variants are CYP2C19*2 (681G/A) and CYP2C19*3 (636G/A), which account for about 99% of all the poor metabolizers (PM) in the Asian population and 87% in the Caucasian population [14]. These variants lead respectively to the creation of a splicing defect and stop codon, and therefore to nonfunctional proteins, resulting in impaired metabolism of CYP2C19 substrates such as Clopidogrel [15]. The substitution of G681A in exon 5 of CYP2C19*2 variant allele creates an aberrant splice site resulting in an alteration of the reading frame of mRNA and consequently a truncated nonfunctional protein. Data from several studies have reported that individuals carrying the CYP2C19*2 allele have impaired pharmacodynamic response to different Clopidogrel doses, as showed with the different platelet function assays used. The CYP2C19*2 allele frequencies vary from ~ 15% among Caucasians and Africans, to ~ 29–35% among Asians [15]. The substitution of G636A in exon 4 of CYP2C19*3 allele results in a premature stop codon, which is common in oriental populations but very rare in Caucasians. Poor metabolizers (PM) of CYP2C19 represent approximately 3–5% of Caucasians. Higher frequencies of PMs (13–23%) are found in most Asian populations [16]. Conversely, CYP2C19*17 allele (c.− 806C>T; rs12248560) was found to be associated with increased CYP2C19 transcription resulting in a modest gain of function. This variant is relatively common, with an average multi-ethnic allele frequency of ~ 3–21%. The study of Alain Li-Wan-Po and co-workers [17] reported that CYP2C19*17 effect is unlikely to have clinical significance except for drugs with very narrow therapeutic indices, such as Clopidogrel. Even then, only mutant homozygous of the variant are likely to be at significantly increased risk.

In 2009, the US Food and Drug Administration (FDA) stated that polymorphisms in *CYP2C19* may be considered as significant and independent predictors of Clopidogrel pharmacokinetics, pharmacodynamics and clinical response [18]. Generally, on the basis of CYP2C19 genotyping, patients are classified as ultra rapid metabolizers (UMs) if they carry double copy of the GOF allele *17 (*17/*17), extensive metabolizers (EMs) if they carry double copy of the wild type allele *1 (*1/*1), intermediate metabolizers (IMs) when carrying one copy of the normal allele and one of the deficient one (*1/*2; *1/*3), or one copy of a deficient allele and one GOF allele (*2/*17, *3/*17), and poor metabolizers (PMs) when carrying double copy of a deficient allele (*2/*2; *3/*3) or two copies of two deficient alleles (*2/*3) [19]. As the Clopidogrel effectiveness depends essentially on its activation by CYP2C19, therapeutic recommendations based on CYP2C19 genotyping were published [15], recommending the use of an alternative antiplatelet agent (prasugrel or ticagrelor, if no contraindication) among PMs and IMs patients, as they have reduced platelet inhibition, increased residual platelet aggregation, increased risk for adverse cardiovascular events [11].

As there is lack of data concerning the genetic bases of Clopidogrel resistance among ACS patients in North African populations in general and Moroccan one specifically, the main objective of our case–control study is to investigate, for the first time in Morocco, whether or not the CYP2C19*2, CYP2C19*3 loss-of-function and CYP2C19*17 gain-of-function alleles are associated with the inter-individual variability of response to Clopidogrel, among a sample of Moroccan ACS patients.

## Main text

### Patients

75 Moroccan ACS patients were recruited from the department of Cardiology, University hospital center Hassan II, Fes, Morocco. Details of patients’ recruitment, study design, DNA extraction and CYP2C19 genotyping are reported in Additional file 1.

### Results

#### Characteristics of the studied population

The studied sample was predominantly composed of around 57 years of age Arabian males (56%; 57 ± 9.72) (Additional file 2: Table S1). Routine pathology data of our patients are represented in (Additional file 3: Table S2): 68.96% of them were under PPI.

#### Verify-now test results vs patients’ baseline characteristics and clinical features

A correlation was held between the verify-now test results (resistant/non-resistant patients) and the patients’ baseline characteristics and clinical features (Additional file 3: Table S3): 100% of the resistant group were female with a statistically significant association with the parameter ‘Gender’ (P = 0.01). A potential trend to a statistical association was also found with ‘Smoking’ and ‘HTA’ parameters (P = 0.07 and 0.09 respectively).

#### Clinical data vs polymorphisms’ distribution

A second correlation was held between patients’ clinical features and the distribution of the 681G>A (*2), 636 G>A (*3) and − 806 C>T (*17) polymorphisms of the CYP2C19 gene (Table 1). Patients with personal antecedents presented higher frequency of the CYP2C19*17 mutant allele than those with no personal antecedents (P = 0.002). No other particular trend was observed (P > 0.05).

**Table 1:** Patients’ clinical data vs polymorphisms distribution

Parameter	CYP2C19*2			P value	CYP2C19*3			P value	CYP2C19*17			P value
	GG %	GA %	AA %		GG %	GA %	AA %		CC %	CT %	TT %	
Familial antecedents				0.8				0.1				0.3
(+)	0	100	0		0	0	100		50	50	0	
(−)	7.1	82.1	10.8		3.9	76.5	19.6		15.4	63.1	21.5	
Personal antecedents				0.1				NC				0.002**
(+)	16.7	72.2	11.1		4.5	68.2	27.3		3.6	57.1	39.3	
(−)	2.5	87.5	10		0	0	0		26.3	65.8	7.9	
Blood pressure				0.3				0.9				0.3
(+)	13	78.3	8.7		4	76	40		16.7	61.1	22.2	
(−)	2.9	85.7	11.4		5.7	77.1	17.2		20.8	70.8	8.3	
Smoking				0.8				0.2				0.8
(+)	8.7	82.6	8.7		8.7	82.6	8.7		16.1	64.5	19.4	
(−)	5.7	82.9	11.4		2.7	73	24.3		12.9	61.3	25.8	
Diabetes				0.1				0.4				0.1
(+)	14.3	71.4	14.3		8	80	12		16.1	58.1	25.8	
(−)	2.7	89.2	8.1		2.9	74.3	22.8		11.5	80.8	7.7	
Dyslipidemia				0.5				0.5				0.1
(+)	25	75	0		11.1	66.7	22.2		14.2	42.9	42.9	
(−)	11.1	77.8	11.1		0	77.8	22.2		10	80	10	

*NC* none calculated

* Statistically significant (Chi square test)

#### ACS subtypes vs polymorphisms’ distribution

Patients were stratified on the basis of the ACS subtype they present (ST+/ST−) and correlated to the polymorphisms distribution, as shown in (Table 2). A statistically significant association between ST+ group and the *2 and *3 mutant alleles (P = 0.002 and 0.04 respectively), but not the *17 one.

**Table 2:** ACS subgroups Vs polymorphisms distribution

Cases						P value (< 0.05)
CYP2C19*2 681G>A	GG %	GA %	AA %	G allele %	A allele %	0.002**
ST (+)	4	80	16	44	56	
ST (−)	9.1	84.85	6.06	51.5	48.5	
CYP2C19*3 636G>A	GG %	GA %	AA %	G allele %	A allele %	0.04*
ST (+)	5.26	73.68	21.06	42.1	57.9	
ST (−)	3.03	75.76	21.21	40.9	59.1	
CYP2C19*17 − 806 C>T	CC %	CT %	TT %	C allele %	T allele %	0.3
ST (+)	30	50	20	55	45	
ST (−)	10.87	69.57	19.56	47	53	

ST (+): Acute Coronary Syndromes with ST segment elevation; ST (−): Acute Coronary Syndromes without ST segment elevation

*Statistically significant (Chi square test)

#### Verify-now test results vs co-presence of the deficient alleles

The same stratification was held this time with the verify-now test results (Additional file 5: Table S4). No particular trend was observed with the alleles taken individually (P > 0.05).

CYP2C19 alleles are inherited as autosomal co-dominant traits, allowing assigning every patient to a Clopidogrel metabolizer phenotype, according to the identified CYP2C19 genotype. In this context, to study the potential synergic effect of the three polymorphisms on Clopidogrel resistance, patients were classified into three groups A, B and C, on the basis of the co-presence of the deficient alleles:

- A group (ultra-rapid metabolizers): *17/*17; *1/*17.
- B group (intermediate metabolizers): *1/*2; *1/*3; *2/*17; *3/*17.
- C group (poor metabolizers): *2/*2; *3/*3; *2/*3.

All the resistant patients were carrying at least one copy of a loss-of-function (LOF) allele of the CYP2C19 gene, with 40% carrying one copy of the *2 or *3 mutant alleles, and 60% with two copies of the LOF alleles *2 and *3; the association was very statistically significant (P = 0.003) (Table 3). Based on CYP2C19 metabolizer profile knowledge and the role of the coded enzyme in Clopidogrel activation, Ums and EMs are expected to have adequate levels of the active metabolite and effective platelet inhibition, while PMs are predicted to have decreased levels of the active metabolite and increased on-treatment platelet aggregation; for IMs, response to Clopidogrel still falling somewhere in between.

**Table 3:** Correlation of the patients’ resistance profile with the co-expression of the three polymorphisms

Verify-now test	Cases groups			P value (< 0.05)
	A group (%)	B group (%)	C group (%)	
Non-resistant	23.6	45.8	30.6	0.003**
Resistant	0	40	60	

* Statistically significant (Chi square test)

#### Allelic and genotypic distribution of the CYP2C19 polymorphisms

As shown in (Additional file 6: Table S5), the three mutant alleles *2; *3 and *17 were the most frequent among patients (51.73, 56.67 and 52% respectively), increasing, thus, the association with these polymorphisms with the pathology.

### Discussion

In the present study, we tried to evaluate the potential modulating effect of *2, *3 and *17 allelic variants of CYP2C19 gene, on Clopidogrel resistance among Moroccan ACS patients. Our results suggested that, taken individually, no one of the three alleles showed statistical association with Clopidogrel resistance (P > 0.05). However, we found a synergic effect between the three alleles, statistically very significant, on Clopidogrel resistance among the treated patients (P = 0.0033).

Several subsequent studies support the positive relationship between CYP2C19 variants and platelet responsiveness to Clopidogrel [20, 21]. The study of Hulot et al. [22], consisting of 28 healthy subjects, followed for 7 days of 75 mg/day of Clopidogrel, showed that, of all the alleles analyzed, only *2 LOF was positively correlated with decreased platelet responsiveness to Clopidogrel. Similarly, Umemura and co-workers performed a study on 47 healthy Asian volunteers, suggesting a determining effect of CYP2C19 pharmacogenomic status on the conversion of Clopidogrel to its active metabolite [23]. Another study called PAPI, for pharmacogenomics of anti-platelet intervention, performed on a sample of 420 healthy Amish volunteers, exposed for 1 week to Clopidogrel, showed that the response to Clopidogrel diverged between the recruited subjects, with 70% of estimated heritability of Clopidogrel response, as the participant were related though a 14 generation pedigree. This inter-individual variability in response between healthy individuals was partially explained by a number of studies suggesting a major effect of CYP2C19 variants, especially *2, on decreased Clopidogrel’ active metabolite formation and increased residual on-treatment platelet reactivity, resulting in poor clinical outcomes and increased risk of complications development [5, 22–32].

Even the association of CYP2C19 polymorphisms with platelet responsiveness to Clopidogrel was largely approved among ACS/PCI patients, the association among patients under Clopidogrel for other indications (e.g. stable angina and arterial fibrillation), have been declared negative [33, 34]. Many authors have explained this inconsistency by the fact that the CYP2C19 effect size on Clopidogrel effectiveness may parallel the effect of this molecule for a given clinical indication; high in patients at high risk for recurrent CVD events in the absence of adequate anti platelet therapy (e.g., ACS/PCI), and low or non-existent for clinical situations where the drug has reduced effect on cardiovascular outcomes (e.g., stable angina, atrial fibrillation and peripheral vascular disease) [27–34]. Thus, CYP2C19 genotyping/Clopidogrel is an example of indication-specific pharmacogenetics [35].

As the Clopidogrel effectiveness depends essentially on its activation by CYP 450 enzymes, especially CYP2C19, therapeutic recommendations based on CYP2C19 genotyping were published: a statement from the US Food and Drug Administration (FDA) reported that Clopidogrel has reduced effectiveness among CYP2C19 PMs, and that, in their case, alternative treatment or therapeutic strategy should be adopted, as they exhibit higher cardiovascular complications rates than do patients having normal CYP2C19 activity [15]. In addition to that, standard doses of Clopidogrel, as found in the product insert, are warranted in ACS/PCI patients predicted to be extensive or ultrarapid metabolizers (i.e., *1/*1, *1/*17, and *17/*17). For patient predicted to be PM (i.e., *2/*2) as well as the IMs ones, the use of an alternative antiplatelet agent (e.g., prasugrel or ticagrelor) was supported by The Clinical Pharmacogenetics Implementation Consortium (CPIC) statement, when not clinically contraindicated [35].

With the adoption of alternative anti-platelet therapies for genetically predisposed patients toward inadequate response, the promise of translating these pharmacogenetic insights into more effective individualized anti-platelet therapy is sparking more excitement and optimism for the future of personalized medicine [18].

Despite a large body of evidence supporting clinical utility, adoption of anti-platelet pharmacogenetics into clinical practice remains slow. Many studies suggest a link between *CYP2C19* polymorphisms and Clopidogrel resistance. This is providing the basis for genotype-informed therapeutic recommendations and optimal individualized antiplatelet treatment, in order to reduce the risk of recurrent CV events and adverse effects such as bleeding, when considering treatment with Clopidogrel.

## Limitations

Our study is the first to investigate the potential association of CYP2C19 polymorphisms with Clopidogrel resistance among ACS patients in Moroccan population. Our results support a role of LOF variants in the CYP2C19 gene as a major marker of Clopidogrel response in Morocco. However, some limitations are to be mentioned, such us the small sample size of patients, the lack of Clopidogrel active metabolite dosing and correlation of SNPs with secondary cardiovascular events. More studies including larger sample sizes and focusing on these points may provide useful help to better understand the phenomenon of Clopidogrel heterogeneity of response.

## Additional files

**Additional file 1: Table S1.** Demographic data of the 75 ACS patients.

**Additional file 2: Table S2.** Routine pathology data of our ACS patients.

**Additional file 3: Table S3.** Baseline characteristics of the ACS patients Vs Verify-Now test results.

**Additional file 4: Table S4.** Verify-Now test results Vs polymorphisms distribution.

**Additional file 5: Table S5.** Genotypic and allelic distribution among the studies sample of patients.

**Additional file 6.** Details of the patients’ recruitment, study design and statistical analysis.

## Abbreviations

ACS: Acute Coronary Syndrome
LOF: loss of function
SNP: single nucleotide polymorphism
RFLP: restriction fragment length polymorphism
