# Supplementary material for: A synergic effect between CYP2C19*2, CYP2C19*3 loss-of-function and CYP2C19*17 gain-of-function alleles is associated with Clopidogrel resistance among Moroccan Acute Coronary Syndromes patients
Source: BMC Res Notes. 2018 Jan 18;11:46. doi: 10.1186/s13104-018-3132-0 (PMC5774088; doi:10.1186/s13104-018-3132-0)
Supplement: Supplementary file 1 — Additional file 1: Table S1. Demographic data of the 75 ACS patients. [file 13104_2018_3132_MOESM1_ESM.docx]

**Table S1: Demographic data of the 75 ACS patients**

| Parameter | Mean ± the standard deviation  or % |
| --- | --- |
|  |  |
| Age (years) | 57.14 ± 9.72 |
| Age of disease’ occurrence (years) | 54.63 ± 10.25 |
| Gender |  |
| Male | 56 % |
| Female | 44 % |
| Ethnicity |  |
| Arab | 87.5 % |
| Berber | 12.5 % |
