# Supplementary material for: A synergic effect between CYP2C19*2, CYP2C19*3 loss-of-function and CYP2C19*17 gain-of-function alleles is associated with Clopidogrel resistance among Moroccan Acute Coronary Syndromes patients
Source: BMC Res Notes. 2018 Jan 18;11:46. doi: 10.1186/s13104-018-3132-0 (PMC5774088; doi:10.1186/s13104-018-3132-0)
Supplement: Supplementary file 2 — Additional file 2: Table S2. Routine pathology data of our ACS patients. [file 13104_2018_3132_MOESM2_ESM.docx]

**Table S2: Routine pathology data of our ACS patients**

| Parameters | Mean ± the standard deviation  or % |
| --- | --- |
|  |  |
| Total Cholesterol (g/l) | 1.86 ± 0.61 |
| HDL (g/l) | 1.24 ± 5.06 |
| LDL (g/l) | 1.24 ± 0.64 |
| Triglycerides (g/l) | 1.53 ± 1 |
| Glucose (g/l) | 1.42 ± 0.8 |
| Creatinine (mg/l) | 10.41 ± 4.27 |
| Fibrinogen | 3.67 ± 1.07 |
| HB (g/dl) | 14.06 ± 2.64 |
| WBc (elts/mm3) | 13102.14 ± 24302.71 |
| Pq (elts/mm3) | 238366.29 ± 103848.5 |
| BMI (Kg/m²) | 26.61 ± 4.13 |
| PPI |  |
| (+) | 68.96% |
| (-) | 31.04% |

**PPI:** proton pomp inhibitors; **BMI:** body mass index; **WBc:** White blood cells; **HB:** hemoglobin; **Pq:** platelets.
