# Supplementary material for: A synergic effect between CYP2C19*2, CYP2C19*3 loss-of-function and CYP2C19*17 gain-of-function alleles is associated with Clopidogrel resistance among Moroccan Acute Coronary Syndromes patients
Source: BMC Res Notes. 2018 Jan 18;11:46. doi: 10.1186/s13104-018-3132-0 (PMC5774088; doi:10.1186/s13104-018-3132-0)
Supplement: Supplementary file 3 — Additional file 3: Table S3. Baseline characteristics of the ACS patients Vs Verify-Now test results. [file 13104_2018_3132_MOESM3_ESM.docx]

**Table S3: Baseline characteristics of the ACS patients Vs Verify-Now test results**

| Parameter | Verify-Now Result | | P Value |
| --- | --- | --- | --- |
|  | **Non Resistants % or means +/- standard deviations** | **Resistants % or means +/- standard deviations** |  |
| Age | 56.66 ± 9.72 | 63.5 ± 9.98 | 0.9 |
| Gender |  |  | **0.01*** |
| Male | 59.2 | 0 |  |
| Female | 40.8 | 100 |  |
| ACS TYPE |  |  | 0.8 |
| ST (+) | 30.2 | 25 |  |
| ST (-) | 69.8 | 75 |  |
| familial antecedents |  |  | 0.7 |
| (+) | 3.2 | 0 |  |
| (-) | 96.8 | 100 |  |
| personal antecedents |  |  | 0.4 |
| (+) | 43.5 | 25 |  |
| (-) | 56.5 | 75 |  |
| Diabetes |  |  | 0.3 |
| (+) | 52.8 | 75 |  |
| (-) | 47.2 | 25 |  |
| Blood pressure |  |  | 0.09 |
| (+) | 57.1 | 100 |  |
| (-) | 42.9 | 0 |  |
| Dyslipidemia |  |  | 0.2 |
| (+) | 54.5 | 100 |  |
| (-) | 45.5 | 0 |  |
| Smoking |  |  | 0.07 |
| (+) | 52.5 | 0 |  |
| (-) | 47.5 | 100 |  |
| Creatinine (mg/l) | 10.45 ± 4.27 | 9.5 ± 4.55 | 0.9 |
| Fibrinogen | 3.59 ± 1.07 | 5.03 ± 1.07 | 0.2 |
| Pq | 242881.35 ± 103248.5 | 149570 ± 83313.62 | 0.4 |
| WBc | 13225.86 ± 24304.72 | 8030 ± 24086.95 | 0.9 |
| Hb | 14.12 ± 2.64 | 12.93 ± 2.06 | 0.2 |
| PPI |  |  | 0.7 |
| used | 67.3 | 75 |  |
| non used | 32.7 | 25 |  |

***:** Statistically significant (chi-square test); **ACS ST (+):** acute coronary syndromes with ST segment elevation; **ACS ST (-):** acute coronary syndromes without ST segment elevation; **WBc:** White blood cells; **HB:** hemoglobin; **Pq:** platelets.
