# Supplementary material for: A synergic effect between CYP2C19*2, CYP2C19*3 loss-of-function and CYP2C19*17 gain-of-function alleles is associated with Clopidogrel resistance among Moroccan Acute Coronary Syndromes patients
Source: BMC Res Notes. 2018 Jan 18;11:46. doi: 10.1186/s13104-018-3132-0 (PMC5774088; doi:10.1186/s13104-018-3132-0)
Supplement: Supplementary file 4 — Additional file 4: Table S4. Verify-Now test results Vs polymorphisms distribution. [file 13104_2018_3132_MOESM4_ESM.docx]

**Table S4: Verify-Now test results Vs polymorphisms distribution**

|  |  |  | Cases |  |  |  | P value  (< 0.05) |
| --- | --- | --- | --- | --- | --- | --- | --- |
| CYP2C19*2  681G>A |  | **GG %** | **GA %** | **AA %** | **G allele** | **A allele** | 0.1 |
|  | **Non-Resistant** | 5.77 | 82.69 | 11.54 | 47.11 | 52.89 |  |
|  | **Resistant** | 20 | 80 | 0 | 60 | 40 |  |
| CYP2C19*3  636G>A |  | **GG %** | **GA %** | **AA %** | **G allele** | **A allele** | 0.3 |
|  | **Non-Resistant** | 5.17 | 75.86 | 18.97 | 43.1 | 56.9 |  |
|  | **Resistant** | 0 | 66.67 | 33.3 | 33.3 | 66.7 |  |
| CYP2C19*17  −806 C>T |  | **CC %** | **CT %** | **TT %** | **C allele** | **T allele** | 0.1 |
|  | **Non-Resistant** | 14.29 | 65.71 | 20 | 47.1 | 52.9 |  |
|  | **Resistant** | 20 | 80 | 0 | 60 | 40 |  |

**681G>A:** G by A substitution polymorphism at position 681; **636G>A:** G by A substitution polymorphism at position 636; **−806 C>T:** C by T substitution polymorphism at position 806. (Chi-square test used).
