# Supplementary material for: A synergic effect between CYP2C19*2, CYP2C19*3 loss-of-function and CYP2C19*17 gain-of-function alleles is associated with Clopidogrel resistance among Moroccan Acute Coronary Syndromes patients
Source: BMC Res Notes. 2018 Jan 18;11:46. doi: 10.1186/s13104-018-3132-0 (PMC5774088; doi:10.1186/s13104-018-3132-0)
Supplement: Supplementary file 5 — Additional file 5: Table S5. Genotypic and allelic distribution among the studies sample of patients. [file 13104_2018_3132_MOESM5_ESM.docx]

Additional Table 5: Genotypic and allelic distribution among the studies sample of patients

|  | Genotypes/alleles | Cases (%) |
| --- | --- | --- |
| CYP2C19*2 |  |  |
|  | GG | 6.89 |
| 681G>A | GA | 82.76 |
|  | AA | 10.35 |
|  | G allele | 48.27 |
|  | A allele | 51.73 |
|  |  |  |
| CYP2C19*3 | GG | 5 |
| 636 G>A | GA | 76.67 |
|  | AA | 18.33 |
|  | G allele | 43.33 |
|  | A allele | 56.67 |
|  |  |  |
| CYP2C19*17 | CC | 14.67 |
| −806 C>T | CT | 66.67 |
|  | TT | 18.66 |
|  | C allele | 48 |
|  | T allele | 52 |

681G>A: G by A substitution polymorphism at position 681; 636G>A: G by A substitution polymorphism at position 636; −806 C>T: C by T substitution polymorphism at position 806.
