# Supplementary material for: A synergic effect between CYP2C19*2, CYP2C19*3 loss-of-function and CYP2C19*17 gain-of-function alleles is associated with Clopidogrel resistance among Moroccan Acute Coronary Syndromes patients
Source: BMC Res Notes. 2018 Jan 18;11:46. doi: 10.1186/s13104-018-3132-0 (PMC5774088; doi:10.1186/s13104-018-3132-0)
Supplement: Supplementary file 6 — Additional file 6. Details of the patients’ recruitment, study design and statistical analysis. [file 13104_2018_3132_MOESM6_ESM.docx]

**Additional File 6:**

**Study population**

Patients’ clinical data (biological parameters and risk factor), demographic characteristics and Verify-Now test results were collected, and an informed consent approved by the Ethical Committee of the University of Hassan II, School of Medicine, Casablanca, was signed by each patient before entering the study. Patients with incomplete clinical information or without Verify-Now test results were excluded from the study. Heparin-free whole blood samples of 4 cc were collected in EDTA tubes from all participants, in order to perform the genetic analysis of CYP2C19 polymorphisms.

**Study design**

We followed the same study protocol as previously described in our published paper [8]: patients were initially made under 300mg loading dose of the generic molecule. If a resistance was noted (PRU> 208), the dose was adjusted to 75mg of Plavix for 7 days (wash out period). a 300mg loading dose of Plavix was prescribed if the PRU remains and the resistance persists; inhibition was considered adequate for good response to treatment if the value reached ≥20%.

We used the Verify-Now test (Accumetrics Inc., San Diego, California) to evaluate platelet function. Two results were reported: The PRU (P2Y12 Reactive Units) and the percent inhibition. The ideal percent of platelet inhibition is ≥30% for Clopidogrel, however, 20-30% inhibition is considered as intermediate response. In our study, resistance to Clopidogrel was defined by <20% inhibition + PRU>208 after many platelet function tests, and non-resistance by ≥20% inhibition + PRU<208.

**DNA extraction**

After collection of whole blood samples from all patients, DNA extraction was performed using the standard method of salting out, as previously described by Miller and co-workers [27].

**CYP2C19 genotyping**

PCR-RFLP reactions were conducted for the CYP2C19*2, CYP2C19*3 and CYP2C19*17 as previously described by [28, 29]. Amplified DNA samples were subjected to restriction digestion by the SmaI, BamHI and NsiI restriction enzymes to identify the *2, *3 and *17 alleles respectively. The digested products were separated on 3% agarose gel electrophoresis stained with 0.5 µg/mL ethidium bromide (BET), and visualized with UV rayons.

**Statistical analysis**

We used SPSS 21.0 software for the statistical part of the study. Hardy–Weinberg Equilibrium test (HWE) was performed for cases and controls groups. To evaluate the statistical significance of association between analyzed variables, we used Chi square test (χ2). Odds ratio (OR) were calculated to estimate the association between genotypes and ACS risk, using a Confidence Interval (CI) of 95%. Significance was approved at P value less than 0.05.
